# Complement MASP-1 Modifies Endothelial Wound Healing

**DOI:** 10.3390/ijms25074048

**Published:** 2024-04-05

**Authors:** Zsuzsanna Németh, Flóra Demeter, József Dobó, Péter Gál, László Cervenak

**Affiliations:** 1 Department of Internal Medicine and Haematology, Semmelweis University, 1085 Budapest, Hungary; nemethzsanna@gmail.com (Z.N.);; 2 Institute of Molecular Life Sciences, HUN-REN Research Centre for Natural Sciences, Hungarian Research Network, 1117 Budapest, Hungary

**Keywords:** MASP-1, endothelial cell, wound healing, angiogenesis, Ca^2+^ response, CREB phosphorylation, adhesion molecules, Matrigel^®^

## Abstract

Endothelial wound-healing processes are fundamental for the maintenance and restoration of the circulatory system and are greatly affected by the factors present in the blood. We have previously shown that the complement protein mannan-binding lectin-associated serine protease-1 (MASP-1) induces the proinflammatory activation of endothelial cells and is able to cooperate with other proinflammatory activators. Our aim was to investigate the combined effect of mechanical wounding and MASP-1 on endothelial cells. Transcriptomic analysis showed that MASP-1 alters the expression of wound-healing-related and angiogenesis-related genes. Both wounding and MASP-1 induced Ca^2+^ mobilization when applied individually. However, MASP-1-induced Ca^2+^ mobilization was inhibited when the treatment was preceded by wounding. Mechanical wounding promoted CREB phosphorylation, and the presence of MASP-1 enhanced this effect. Wounding induced ICAM-1 and VCAM-1 expression on endothelial cells, and MASP-1 pretreatment further increased VCAM-1 levels. MASP-1 played a role in the subsequent stages of angiogenesis, facilitating the breakdown of the endothelial capillary network on Matrigel^®^. Our findings extend our general understanding of endothelial wound healing and highlight the importance of complement MASP-1 activation in wound-healing processes.

## 1. Introduction

The complement system is an important part of our innate immune system, acting as a first-line defense in our blood. It can be activated by three different pathways: the classical, lectin, and alternative pathways. Mannose-binding lectin (MBL); ficolin-H, -L, and -M; and collectins 10 and 11, the pattern recognition molecules of the lectin pathway, circulate in the blood in complexes with associated serine proteases and recognize pathogens or altered host cells [1,2]. Recognition leads to the activation of MBL-associated serine proteases 1 and 2 (MASP-1 and -2) [3].

MASP-1 is the most abundant enzyme of the lectin pathway, and, in addition to cascade activation by the cleavage of MASP-2, it directly activates endothelial cells through the cleavage of protease-activated receptors (PARs). The activation of PAR-1, -2, and -4 upon cleavage by MASP-1 leads to the activation of several signaling pathways (Ca^2+^, NF-κB, MAPK, CREB, and JNK) and proinflammatory genes [4] in human umbilical vein endothelial cells (HUVECs) [5,6]. As a result, MASP-1 induces a proinflammatory phenotype, which is characterized by the increased expression of proinflammatory cytokines [6] and adhesion molecules, enhanced adhesion between ECs and neutrophils [7], and increased permeability [8]. MASP-1 is able to cooperate with several other proinflammatory activators (e.g., LPS, histamine, IFNγ, and bradykinin) to enhance endothelial cell activation [9]. Hypoxia has also been shown to enhance the effect of MASP-1 on endothelial cells [10]. In these experiments, we used 2 μM of MASP-1 to study the individual effects and a lower (suboptimal) dose (0.6 μM) to test the possible interactions with other activating factors. 

Maintaining and restoring the integrity of the circulatory system is of fundamental importance for all body functions. Due to their location, endothelial cells (ECs) are involved in all major phases of wound healing: initial clot formation/fibrinolysis, inflammation, and the final phase of cell proliferation and tissue remodeling [11]. The inflammatory phase is pivotal in the wound-healing process, not only by encountering the invading microbes or new tissue constituents but also by actively contributing to tissue repair processes [12]. 

The involvement of the complement lectin pathway in wound-healing processes has been actively investigated in the context of atherosclerosis and atherosclerosis-related diseases [13,14] as well as in the healing of chronic wounds [15]. However, limited data are available on the effect of lectin pathway activation on normal wound-healing processes in otherwise healthy individuals. Elevated levels of complement components after trauma indicate extensive complement activation [16] and complement-modulatory therapies in animal studies have shown a protective role after a traumatic event [17].

Here, we show for the first time how complement MASP-1 modulates endothelial cell involvement in wound-healing processes. 

## 2. Results

### 2.1. rMASP-1 Alters the Expression of Wound-Healing-Related and Angiogenesis-Related Genes

We have previously shown that rMASP-1 significantly alters the expression of inflammation- and permeability-related genes [4,8]. Using the same mRNA expression database (all data are available in the NCBI Gene Expression Omnibus database under the series accession number GSE98114), we now investigated whether rMASP-1 affects the expression of angiogenesis- and wound-healing-related genes. We used the Gene Ontology Annotation (UniProt-GOA) database and searched for human genes from the categories ‘GO:0001525 angiogenesis’ (336 genes) and ‘GO:0042060 wound healing’ (323 genes). Gene set enrichment analysis (GSEA) examines whether a given set of genes is represented as higher or lower in the ranked list of fold changes than the average fold changes of all genes. We found that both angiogenesis-related (normalized enrichment score (NES): 2.16) and wound-healing-related (NES: 1.62) genes were overrepresented at the top of the ranked list of genes after rMASP-1 treatment, which reflects a significant upregulation of both wound-healing-related and angiogenesis-related gene sets in response to rMASP-1 (Figure S1a,b). We also observed significant enrichment (NES: 2.1) (Figure S1c) when the two categories were combined (603 genes).

Based on these findings, we further investigated the wound-healing process of the HUVEC cells, with particular attention to the involvement of MASP-1. 

### 2.2. Calcium Wave Propagation 

**Figure 1:**
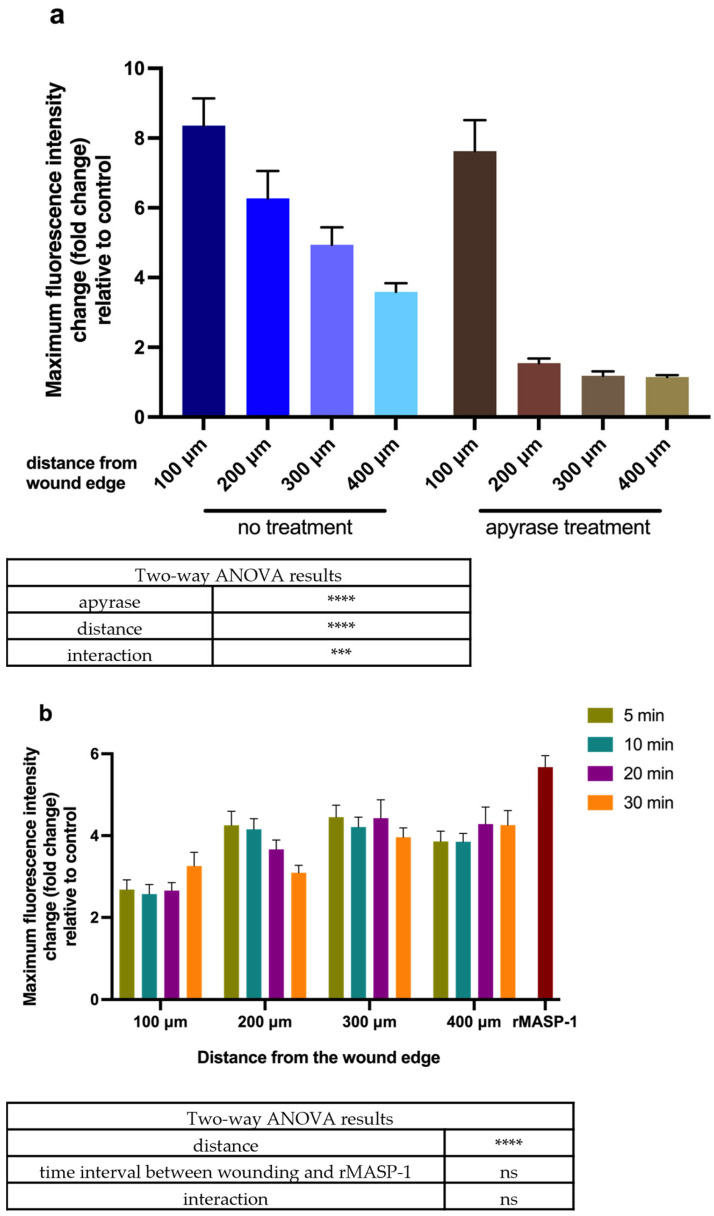
Ca^2+^ mobilization in response to mechanical wounding and rMASP-1: (**a**) Confluent layers of HUVECs were cultured in 96-well plates, and then cells were loaded with 2 μM of Fluo-4-AM. Sequential images were taken every 5 s using fluorescence microscopy. Initially, two images were taken to determine baseline fluorescence, and then HUVEC layers were scratched with a sterile pipette tip. The response was measured for 2 min. Apyrase treatment (10 U/mL) was applied 5 min before the measurement. (**b**) Cells were loaded with 2 μM of Fluo-4-AM and then scratched using a sterile pipette tip. Next, 0.6 μM of rMASP-1 treatment was added to the wells 5, 10, 20, or 30 min after scratching; then, sequential images were taken every 5 s using fluorescence microscopy. The effect of 0.6 μM of rMASP-1 without scratching was also measured for comparison. (**a**,**b**) Distances were measured from the edge of the initial wound. Two-way ANOVA was used for statistical analysis. ****: *p* < 0.0001; ***: *p* < 0.001; ns: not significant.

As previously shown by others, the mechanical stimulation of endothelial cells induces a calcium wave that continues to propagate [18]. We could also observe this by scratching the HUVEC layer using a sterile pipette tip. This response of the endothelium layer was rapid, initiated within seconds after wounding, and propagated in a wave-like manner to neighboring cells (Video S1). The intensity of Ca^2+^ mobilization decreased with increasing distance from the initial wound. ATP scavenger apyrase did not inhibit Ca^2+^ mobilization in cells closest to the scratch but inhibited the propagation of the calcium wave from one cell to another (Figure 1a, Video S2). As shown previously, rMASP-1 is able to initiate Ca^2+^ mobilization in HUVECs [5]; moreover, this signaling process can be synergistically modified by other Ca^2+^-mobilizing factors, such as bradykinin or histamine [9] as well as hypoxia [10]. We investigated whether Ca^2+^ mobilization induced by mechanical wounding could also modify the Ca^2+^-inducing potential of rMASP-1. For these experiments, we used a suboptimal dose (0.6 μM) of rMASP-1 to ensure the accurate measurement of the cellular response. When rMASP-1 treatment was preceded by mechanical wounding, rMASP-1-induced Ca^2+^ mobilization was slightly inhibited, which was most pronounced in cells closest to the wound and did not change over the time period studied (30 min) (Figure 1b).

### 2.3. Changes in CREB Phosphorylation and NFκB Activation 

We measured CREB phosphorylation and NFκB activation (localization in the nucleus) in response to mechanical wounding and investigated the impact of rMASP-1 on these processes. 

**Figure 2:**
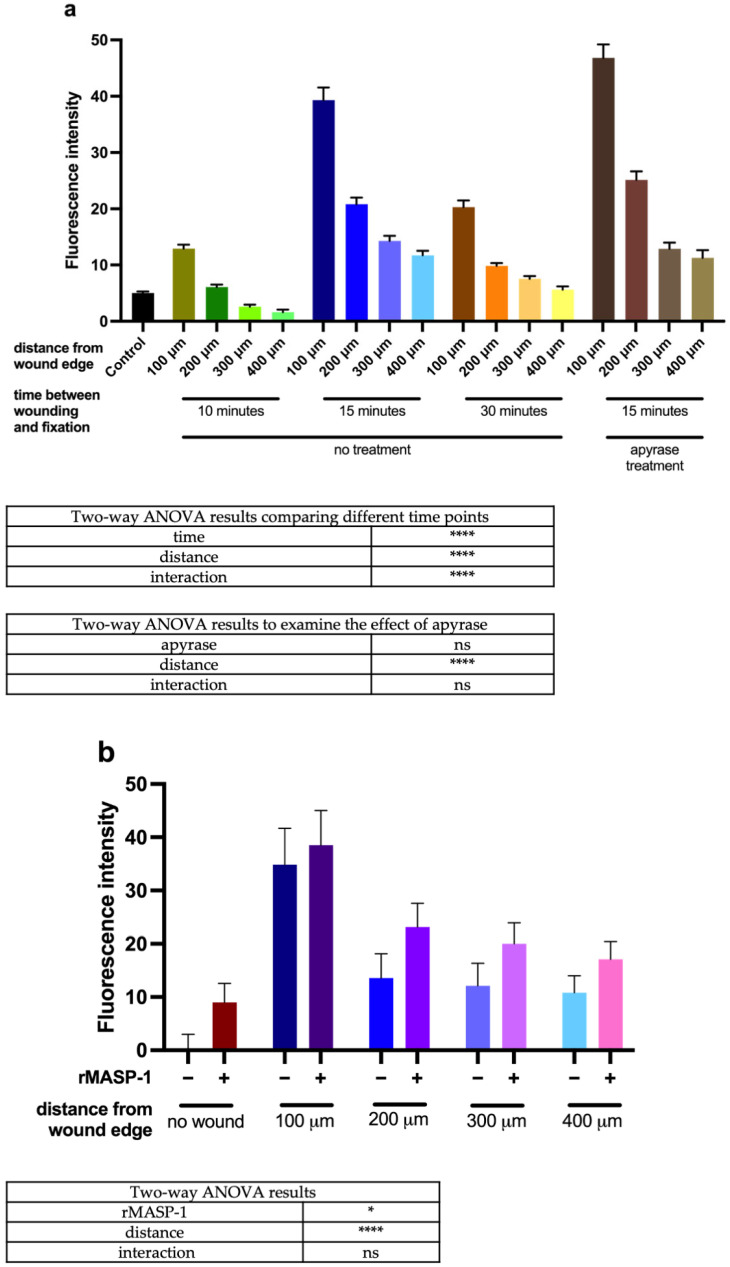
Changes in CREB phosphorylation in response to wounding and rMASP-1: (**a**) Confluent layers of HUVECs were cultured in 96-well plates and scratched using a sterile pipette tip to create a wound. Apyrase treatment (10 U/mL) was applied 5 min before the wounding. Cells were fixed with ice-cold methanol–acetone (1:1) solution 10, 15, or 30 min after scratching. (**b**) The wells were scratched using a sterile pipette tip, and after 5 min, 0.6 μM of rMASP-1 was added to the wells. Cells were fixed with ice-cold methanol–acetone (1:1) 15 min after rMASP-1 treatment. (**a**,**b**) Cells were labeled with rabbit anti-human phospho-CREB antibody (1:200) and stained with goat anti-rabbit Alexa568 (1:500) and Hoechst (1:50,000) nuclear staining. Images were taken using an Olympus IX-81 inverted fluorescence microscope, and the mean intensity of red fluorescence in the nuclear region was evaluated using CellP 3.4 software (Olympus Soft Imaging Solutions GmbH, 2011). Distances were measured from the edge of the initial wound. Two-way ANOVA was used for statistical analysis. ****: *p* < 0.0001; *: *p* < 0.05 ns: not significant.

CREB phosphorylation was measured 10, 15, or 30 min after wounding, and the strongest response was observed after 15 min (Figure 2a). Pretreatment with apyrase did not affect the wounding-induced phosphorylation of CREB. As previously shown, rMASP-1 induces CREB phosphorylation [5]. When mechanical wounding was followed immediately (5 min) by 15 min of rMASP-1 treatment, CREB phosphorylation was more pronounced than either the effect of rMASP-1 or wounding alone. The potentiating effect of rMASP-1 was independent of the distance to the wound edge (Figure 2b).

We did not find any changes in NFκB localization 15, 30, 60, or 90 min after wounding (Figure S2).

### 2.4. Expression of Adhesion Molecules 

**Figure 3:**
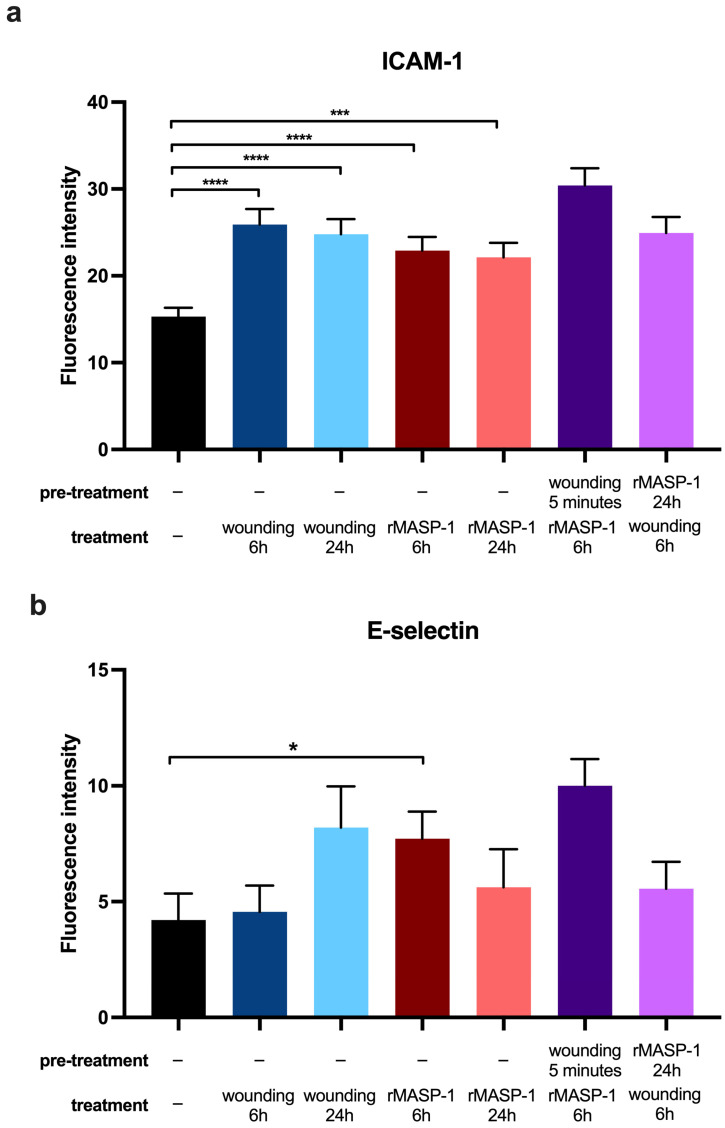
Changes in adhesion molecule expression in response to wounding and rMASP-1. Confluent layers of HUVECs were cultured in 96-well plates, and rMASP-1 pretreatment (0.6 μM) was applied to some of the wells. The wound was made using a sterile pipette tip. Cells were fixed after 6 or 24 h and then labeled with mouse anti-human ICAM-1 (**a**) or mouse anti-human E-selectin (**b**) antibodies (1:500) and stained with goat anti-mouse Alexa568 (1:500) and Hoechst (1:50,000) nuclear staining. Images were taken using an Olympus IX-81 inverted fluorescence microscope, and the mean intensity of red fluorescence in the cytoplasm was evaluated using CellP 3.4 software (Olympus Soft Imaging Solutions GmbH, 2011). ****: *p* < 0.0001; ***: *p* < 0.001; *: *p* < 0.05.

**Figure 4:**
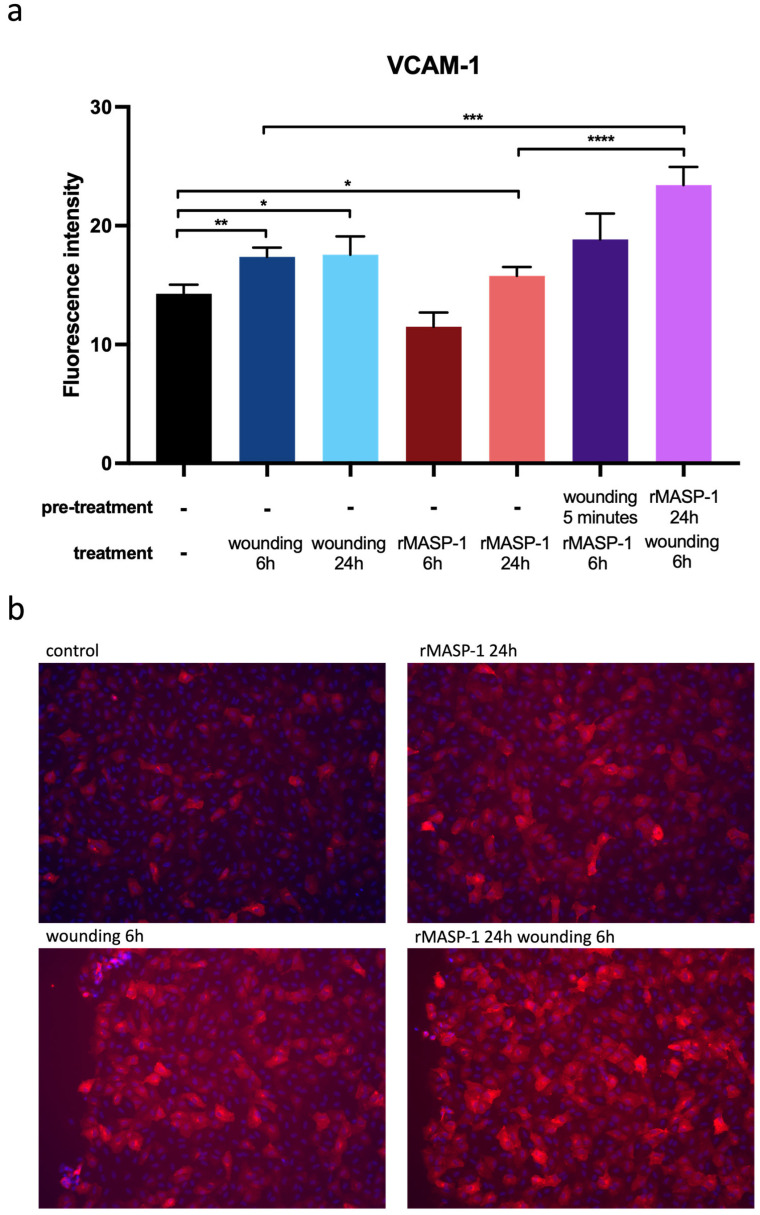
Changes in VCAM-1 expression in response to wounding and rMASP-1. Confluent layers of HUVECs were cultured in 96-well plates, with rMASP-1 pretreatment (0.6 μM) applied to some of the wells. The wound was made using a sterile pipette tip. Cells were fixed after 6 or 24 h and then labeled with mouse anti-human VCAM-1 (1:500) and stained with goat anti-mouse Alexa568 (1:500) and Hoechst (1:50,000) nuclear staining. (**a**) Images were taken using an Olympus IX-81 inverted fluorescence microscope, and the mean intensity of red fluorescence in the cytoplasm was evaluated using CellP 3.4 software (Olympus Soft Imaging Solutions GmbH, 2011). Panel (**b**) shows representative images from three independent experiments. ****: *p* < 0.0001; ***: *p* < 0.001; **: *p* < 0.01; *: *p* < 0.05.

We used fluorescence microscopy to investigate the expression changes in three well-known adhesion molecules, namely E-selectin, intercellular adhesion molecule 1 (ICAM-1), and vascular cell adhesion molecule 1 (VCAM-1), 6 or 24 h after wounding. Wounding induced weak but significant ICAM-1 and VCAM-1 expression after 6 and 24 h (Figure 3 and Figure 4). Although we could observe the induction of E-selectin expression 24 h after wounding, it did not reach statistical significance (*p* = 0.0504). A suboptimal dose of rMASP-1 induced weak but significant ICAM-1 expression after 6 and 24 h, VCAM-1 expression after 24 h, and E-selectin expression after 6 h (Figure 3 and Figure 4). When HUVECs were pretreated with rMASP-1 for 24 h followed by scratching, VCAM-1 expression was further induced after 6 h (Figure 4).

### 2.5. Wound-Healing Assay

**Figure 5:**
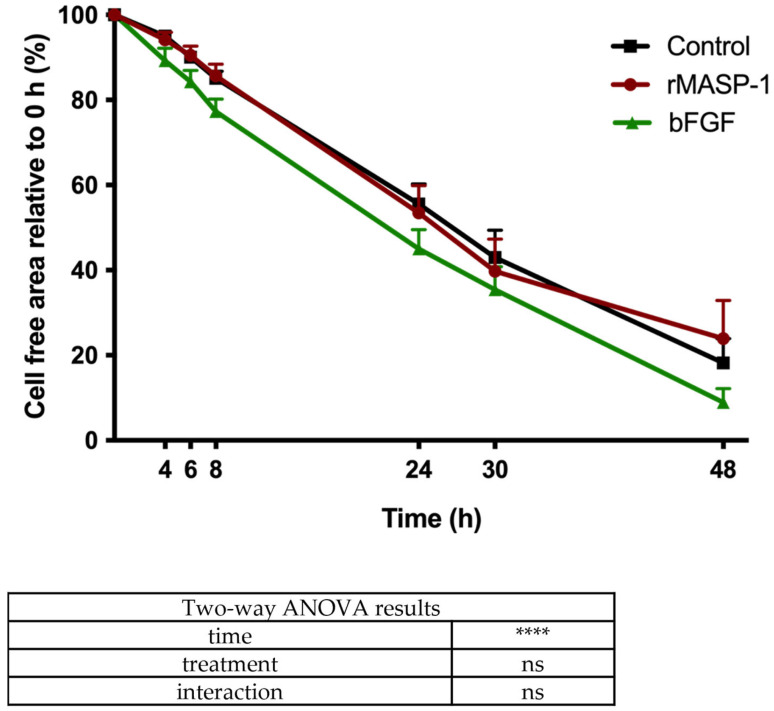
Effect of rMASP-1 on wound closure rate. HUVEC cells were cultured in Ibidi 3-well culture inserts (which created a 500 μm wide cell-free area) until confluence, after which the inserts were removed, and the areas were photographed for 48 h. rMASP-1 (2 μM) or bFGF (1.6 ng/mL) was administered immediately after the removal of the inserts. We determined the size of the cell-free area using ImageJ software (1.54d) and plotted it against the 0 h time point. Two-way ANOVA was used for data analysis. ****: *p* < 0.0001; ns: not significant.

We conducted a wound-repair assay to study the rate of wound closure. To measure the individual effect of rMASP-1 in these experiments, we used 2 μM of it. We found that rMASP-1 did not significantly affect the wound closure rate of HUVECs. The basic fibroblast growth factor (bFGF), which was used as a positive control, slightly but not significantly increased the wound closure rate (Figure 5).

### 2.6. Capillary Networks on Matrigel^®^

**Figure 6:**
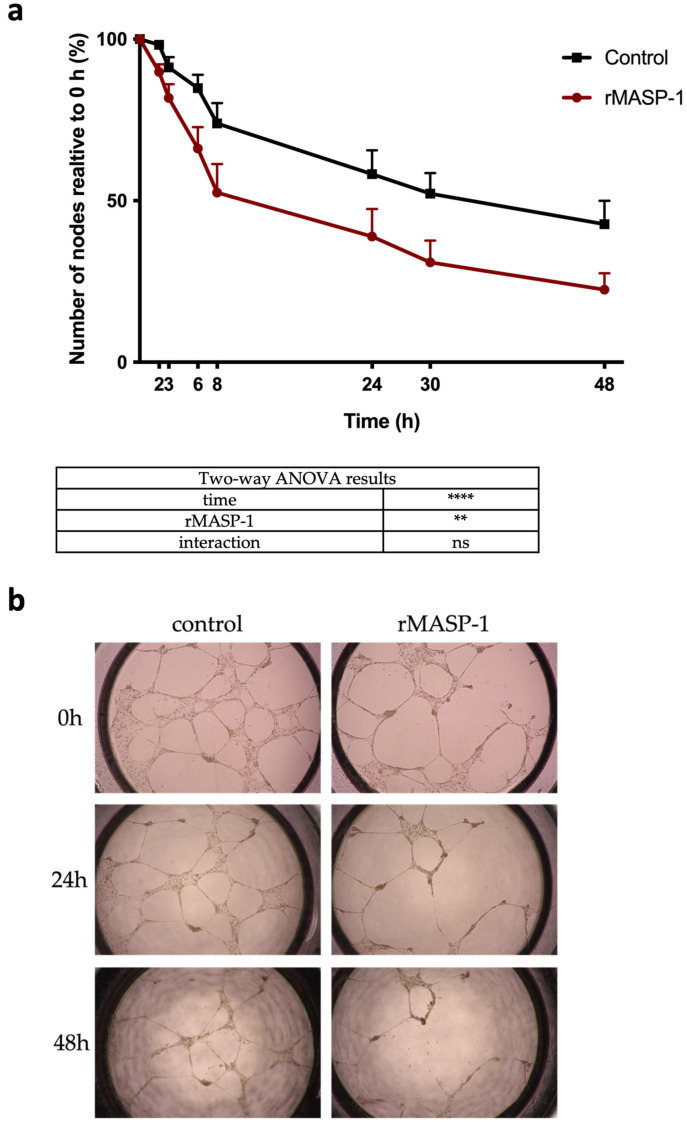
Effect of rMASP-1 on capillary network integrity. HUVECs were seeded onto 15-well “Angiogenesis µ-Slides” coated with Matrigel^®^ in 100% confluence and cultured for 16 h until typical capillary-like network structures were formed. The cells were then treated with 2 μM of rMASP-1. Photographs were taken 0, 2, 3, 5, 6, 8, 24, 30, and 48 h after treatment. (**a**) The number of nodes was determined using ImageJ software (1.54d) and then plotted against the number of nodes before treatment. Two-way ANOVA was utilized for data analysis. Panel (**b**) shows representative images from three independent experiments. ****: *p* < 0.0001; **: *p* < 0.01; ns: not significant.

When seeded on Matrigel^®^, endothelial cells formed a typical capillary-like network. rMASP-1 treatment significantly accelerated the disintegration of these networks compared to the no-treatment control (Figure 6).

## 3. Discussion

**Figure 7:**
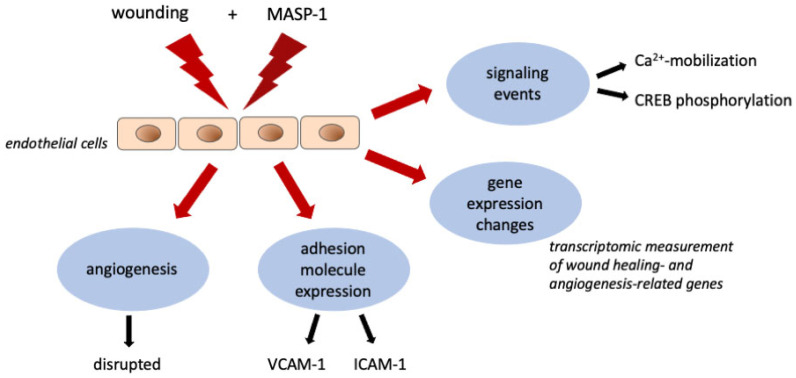
Complement MASP-1 modifies endothelial wound healing. Our results demonstrate that the activation of MASP-1 modifies the response of endothelial cells to mechanical wounding at several levels: signaling events, transcriptomic changes, adhesion molecule expression, and complex functional outcomes (e.g., angiogenesis).

In this study, we found that complement MASP-1 modifies endothelial cell involvement in wound-healing processes. At the transcriptomic level, MASP-1 significantly modulated the expression of wound-healing-related and angiogenesis-related genes. Wound-healing-induced CREB phosphorylation was further enhanced, and a second reduced Ca^2+^ wave was triggered if MASP-1 was present. MASP-1 promoted the disintegration of the endothelial network on Matrigel^®^ and potentiated the VCAM-1 expression-inducing effect of wounding (Figure 7). 

The identification of signals that trigger endothelial wound-healing responses is still a matter of active investigation. Immediately after wounding, endothelial cells develop a Ca^2+^ wave that propagates from the border of the wound toward the more distant areas of the monolayer. This phenomenon has been described in bovine corneal endothelial cells (BCENs) as well as in HUVECs [19,20]. In BCENs, Ca^2+^-wave propagation was inhibited by apyrase treatment [19], whereas in HUVECs, the picture is a little more complicated. Contrary to our results, laser-induced Ca^2+^-wave propagation was not inhibited by apyrase [21], which may be caused by different induction methods (laser vs. mechanical). Pohl et al. found that in addition to apyrase, a gap junction blocker was also required to completely block the Ca^2+^ wave, which was triggered by a mechanical stimulus, but in the presence of apyrase, only some of the neighboring cells took up the Ca^2+^ wave [22]. We confirmed that mechanical stimulation induced a Ca^2+^ wave in HUVECs and found that cells closest to the wound gave a Ca^2+^ signal in the presence of apyrase, but the Ca^2+^-wave propagation was inhibited. As we showed earlier, MASP-1 initiates Ca^2+^ mobilization in HUVECs [5]. When MASP-1 treatment followed mechanical wounding, a secondary Ca^2+^ mobilization was induced, which was smaller than the effect of MASP-1 alone, and the mild inhibition persisted for 30 min, especially in the vicinity of the wound. 

Another potential signal that regulates the expression of several genes is CREB phosphorylation. Togo found in Madin–Darby canine kidney cells (MDCKs) that mechanical wounding induces CREB phosphorylation in the wounded and neighboring cells [23], and CREB phosphorylation was also induced in Swiss 3T3 fibroblasts [24]. Here, we showed for the first time that mechanical wounding induces CREB phosphorylation in human endothelial cells. We have demonstrated earlier that MASP-1 also induces CREB phosphorylation [5], and in the current study, we showed that wounding and MASP-1 stimulus synergistically induce CREB phosphorylation. This suggests an enhanced inflammatory response at the endothelial cell level when microbial invasion is accompanied by tissue damage. 

The activation of the NFκB pathway is a well-known participant in the regulation of inflammation that often accompanies the wound-healing process. In the rat and mouse aorta, 45 min after limited endothelial denudation, Collins et al. found NFKB activation in endothelial cells at the leading edge [25]. In contrast, in human dermal endothelial cells, IκBα, the inhibitory subunit of NFκB, was activated following wounding [26]. In line with this, we could not detect any NFκB activation in HUVECs in response to mechanical damage.

The expression of various adhesion molecules is involved in the leukocyte/endothelial cell interaction step of wound healing. ICAM-1, expressed by endothelial cells, is best known to regulate leukocyte recruitment from circulation. Nagaoka et al. found that ICAM-1 is expressed on the surface of endothelial cells in the wounded skin of mice, and the loss of ICAM-1 inhibited the wound-healing process [27]. ICAM-1 is also expressed on the surface of endothelial cells in injured human skin [28]. Consistent with these findings, we have shown that ICAM-1 expression is induced by mechanical wounding on HUVECs, and its expression remains high even after one day after wounding.

In addition to ICAM-1, VCAM-1 also mediates the vascular adhesion and the transendothelial migration of leukocytes [29]. In the rat and mouse aorta after limited endothelial denudation, Collins et al. found localized VCAM-1 expression, restricted to endothelial cells immediately adjacent to the wound edge [25]. Müller et al. found that 18% of the endothelial cells in intact skin expressed VCAM-1 and only at low intensity, whereas 51% of the endothelial cells in injured skin were positive at higher intensities. They found the strongest expression 4–6 h after wounding [30]. This is in agreement with our results; we also found elevated VCAM-1 levels in HUVECs 6 h after wounding. In our previous studies at the transcriptomic level, we found elevated VCAM-1 mRNA expression after MASP-1 treatment [4], but surface VCAM-1 expression showed large individual variance [7,10]. In this study, we showed that when cells were previously treated with MASP-1, mechanical wounding induced stronger VCAM-1 expression than wounding alone. It is possible that MASP-1 treatment elevates the VCAM-1 mRNA levels, and a second stimulus (wounding) results in elevated protein expression. 

A general characteristic of the success of the wound-healing process is the time required for wound closure. Svensson et al. found that neither PAR-1 nor PAR-2 agonists increased the rate of wound healing in HUVECs [31], and wound closure in PAR-1-deficient mice was normal [32]. Consistent with these results, we found that MASP-1, using PAR-1, PAR-2, and PAR-4 signaling pathways in HUVECs, did not affect the rate of wound closure at the endothelial cell level.

During the proliferation phase of wound healing, the integrity of the capillary network is restored with suitable angiogenesis. It has been described that thrombin and PAR-1 activator peptide inhibited endothelial tube formation, whereas PAR-2 activator peptide had no effect on it [33,34]. In this study, we showed that MASP-1 accelerates the disintegration of the endothelial network.

The involvement of complement components in wound healing is still under extensive research. For now, it is known that complement activation is needed for normal tissue repair. Both C3 and C5 have been recently shown to accelerate and increase acute wound healing [35]. It has also been shown that C1q is deposited in wound-healing skin independent of complement activation and topically applied C1q showed proangiogenic activity [36]. In “normal” wound healing, the inflammatory phase is resolved within a few days. However, inappropriate complement activation (as can be seen in chronic wounds or burn wounds) will result in detrimental effects, promoting prolonged inflammation and cell death [15,35]. MAC deposition is found in the majority of chronic leg ulcers [37], and enhanced levels of C3d have been shown in burn wounds until 46 days after burn trauma [38]. In animal models of burn wounds, the application of a C1 inhibitor (which inhibits several proteases of the fibrinolytic, clotting, kinin, and complement pathways, including MASP-1) reduced edema formation and inflammation-induced tissue destruction [38].

The activation of MASP-1 during wound-healing processes can be a part of a normal tissue repair process (activated by damaged/altered host cells) or a result of an infection. Duis et al. have shown in skin biopsies that incision alone leads to a limited form of inflammation, but inflammation is more pronounced when accompanied by infection [39]. This is consistent with our results, where we could observe a second Ca^2+^ response, stronger CREB phosphorylation, and VCAM-1 expression when both wounding and MASP-1 were present. In addition, MASP-1 slowed down the subsequent angiogenesis. 

In conclusion, our findings expand our general knowledge of endothelial wound healing and highlight the importance of complement MASP-1 activation in acute and chronic wound-healing processes.

## 4. Materials and Methods

### 4.1. Reagents

The recombinant catalytic fragment of human MASP-1 (CCP1-CCP2-SP, here referred to as rMASP-1) was expressed in *Escherichia coli*, refolded and purified using the method of Dobó et al. in the absence of inhibitors, with modifications as previously described [40,41]. rMASP-1 preparations were free from bacterial contaminations and could be inhibited by the C1 inhibitor as previously described [7,42,43]. 

All other reagents were purchased from Merck–Sigma-Aldrich, unless otherwise stated.

### 4.2. Preparation and Culturing of Human Umbilical Vein Endothelial Cells (HUVECs) 

Endothelial cells were harvested from fresh umbilical cords obtained during the normal delivery of healthy neonates by collagenase digestion as described earlier [6]. HUVECs were grown in gelatin-coated flasks (Corning^®^ Costar^®^, Corning NY, USA) in an MCDB131 medium (ThermoFisher Scientific, Waltham, MA, USA) completed with 5% heat-inactivated calf serum (FCS), 2 ng/mL human recombinant epidermal growth factor (R&D Systems, Minneapolis, MN, USA), 1 ng/mL human recombinant basic fibroblast growth factor, 0.3% insulin transferrin selenium (ThermoFisher Scientific), 1% chemically defined lipid concentrate (ThermoFisher Scientific), 1% Glutamax solution (ThermoFisher Scientific), 1% penicillin–streptomycin antibiotic solution, 5 μg/mL ascorbic acid, 250 nM hydrocortisone, 10 nM HEPES, and 7.5 U/mL heparin. For some experiments, the medium was replaced with AIM-V medium (ThermoFisher Scientific) completed with 1% FCS, 2 ng/mL human recombinant epidermal growth factor (R&D Systems), 1 ng/mL human recombinant basic fibroblast growth factor, and 7.5 U/mL heparin. 

All experiments were performed in at least 3 independent, primary HUVEC cultures obtained from different individuals before passage 5.

### 4.3. Gene Set Enrichment Analysis (GSEA)

We used the same microarray database as in our previous article [4]. The classical GSEA analysis was performed using GSEA version 4.3.2 from the Broad Institute (MIT) [44]. Normalized enrichment scores (NESs), nominal *p* value, and FDR q value were calculated. The data are available in the NCBI Gene Expression Omnibus database under the accession number GSE98114.

### 4.4. Intracellular Ca^2+^ Mobilization Assay

Intracellular Ca^2+^ mobilization was measured using the method previously described. Cells were seeded in 96-well plates at 100% confluence and cultured in an MCDB131 medium for 24 h, and then the medium was replaced with AIM-V for an additional 24 h. Cells were loaded using 2 μM of Fluo-4-AM (ThermoFisher Scientific) for 20 min and incubated in HBSS for another 20 min. If necessary, apyrase treatment (10 U/mL) was added to the cells for 10 min before measurements. Measurements were performed using fluorescence microscopy, and sequential images were taken every 5 s. To determine baseline fluorescence, two photographs were taken before adding the treatment or scratching the cells. Cell-layer scratching was performed using a sterile pipette tip. For some measurements, 0.6 μM of rMASP-1 was added to the wells 5, 10, 20, or 30 min after scratching. For each distance (100 μm, 200 μm, 300 μm, and 400 μm), at least twenty cells were analyzed using CellP software (version 5.2). 

### 4.5. Measurement of CREB Phosphorylation and NFκB Activation by Fluorescent Microscopy

Cells were seeded in 96-well plates at 100% confluence and cultured for 2 days. Apyrase treatment (10 U/mL) was added to some wells for 10 min prior to scratching. The monolayer of cells was scratched using a sterile pipette tip 10, 15, and 30 min (CREB phosphorylation) or 15, 30, and 60 min (NFκB activation) before fixation. Some wells were treated with 0.6 μM of rMASP-1 after scratching. Cells were fixed in ice-cold methanol–acetone (1:1) for 10 min. Cells were stained with rabbit anti-human phospho-CREB (1:200 Cell Signaling Technology Inc., Danvers, MA, USA) antibody or rabbit anti-human NFκB p65 (1:200, Santa Cruz Biotechnology, Dallas, TX, USA) antibody followed by Alexa568 conjugated goat anti-rabbit IgG (1:500) and Hoechst 33342 (1:50,000, Invitrogen, Waltham, MA, USA) (Table 1). Photographs were taken using an Olympus IX-81 fluorescence microscope. All analyses were performed using the original unmodified images using CellP software (version 5.2). Nuclear mean red fluorescence (pCREB) or the ratio of the cytoplasmic and nuclear mean red fluorescence (NFκB) was calculated.

### 4.6. Wound-Healing Assay

Confluent layers of endothelial cells were seeded and cultured in 96-well plates for 2 days. The wound was made manually by scratching the monolayer of the cells using a sterile pipette tip. Then, bFGF (1.6 ng/mL) or rMASP-1 (2 μM) treatment was applied. Photographs were taken after 0, 2, 4, 6, 24, 30, and 48 h using a microscope with a relative objective of 20x. The analysis of the remaining wound area in each image was performed using ImageJ software (version 1.54e).

### 4.7. Capillary Network Integrity—Matrigel^®^ Assay

HUVECs were seeded in 15-well “Angiogenesis µ-Slides” at 100% confluence and cultured for 16 h until typical network-like structures were formed. Next, 2 μM of rMASP-1 was added to the cells. Wells were photographed 0, 2, 3, 6, 8, 12, 24, 30, and 48 h after treatment. The number of branch points was measured using ImageJ software (version 1.54e) [45].

### 4.8. Visualization of Adhesion Molecules by Fluorescent Microscopy

Confluent layers of endothelial cells were seeded and cultured in 96-well plates for one day. The monolayer of cells was scratched 24, 6, or 2 h before fixation. Some wells were treated with 0.6 μM of rMASP-1. Cells were fixed in ice-cold methanol–acetone (1:1) for 10 min. Then, cells were stained with primary anti-human antibodies against E-selectin, ICAM-1 or VCAM-1 (as indicated in Table 1) followed by Alexa568-conjugated goat anti-mouse IgG (1:500) and Hoechst 33342 (1:50,000, Invitrogen). Images were taken using an Olympus IX-81 fluorescence microscope. 

### 4.9. Statistical Analysis

Experiments were performed in duplicates (Ca^2^+ mobilization assay, phosphor-CREB and NFκB activation measurement, and adhesion molecule visualization) or triplicates (wound-healing and Matrigel^®^ assays) and repeated at least three times using HUVECs from different individuals. Statistical analysis was performed after evaluating normality using Student’s *t*-test or two-way ANOVA with GraphPad Prism 10 software. A *p* ≤ 0.05 was considered statistically significant. Data are presented as means ± SEM unless otherwise stated. 

## Figures and Tables

**Table 1:** Antibodies used for fluorescent microscopy.

Antibody	Producer	Catalog Number	Dilution
Mouse anti-human E-selectin	Invitrogen	BMS110	1:500
Mouse anti-human ICAM-1	Invitrogen	BMS108	1:500
Mouse anti-human VCAM-1	BD Pharmingen	555,645	1:500
Rabbit anti-human NFκB p65	Santa-Cruz Biotechnology	sc-372	1:200
Rabbit anti-human phosphor-CREB	Cell Signaling Technology Inc.	9198	1:200
Alexa568 conjugated goat anti-mouse IgG	Invitrogen	A-11004	1:500
Alexa568 conjugated goat anti-rabbit mouse IgG	Invitrogen	A-11036	1:500

## Data Availability

Datasets generated during and/or analyzed in the current study are available from the corresponding author upon reasonable request.

## Supplementary Materials

The following supporting information can be downloaded from https://www.mdpi.com/article/10.3390/ijms25074048/s1.
