# Supplementary material for: Complement MASP-1 Modifies Endothelial Wound Healing
Source: Int J Mol Sci. 2024 Apr 5;25(7):4048. doi: 10.3390/ijms25074048 (PMC11012537; doi:10.3390/ijms25074048)
Supplement: Supplementary file 1 [file ijms-25-04048-s001.zip › supplementary material.docx]

**Complement MASP-1 modifies endothelial wound healing**

**Zsuzsanna Németh^1^, Flóra Demeter^1^, József Dobó^2^, Péter Gál^2^, László Cervenak^1,*^**

^1^ Research Laboratory, Department of Internal Medicine and Hematology, Semmelweis University, Budapest, Hungary

^2^Institute of Enzymology, HUN-REN Research Centre for Natural Sciences, Hungarian Research Network, Budapest, Hungary

**Supplementary material**


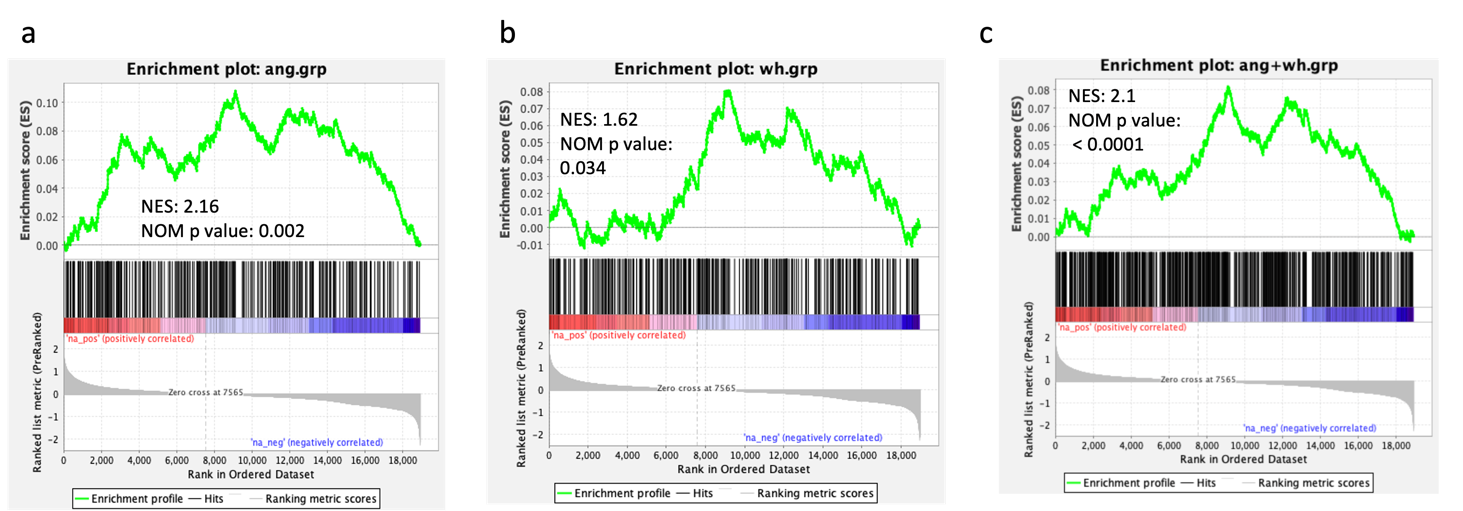


**Figure S1. Gene set enrichment analysis (GSEA) showing significant enrichment of angiogenesis- and wound healing-related genes**

GSEA were run on the database containing data from rMASP-1 treated and not treated HUVECs (available in the NBI Gene Expression Omnibus database, accession number: GSE98114). Genes were retrieved from the Gene Ontology Annotation database. Panel a: GO:0001525 angiogenesis; panel b: GO:0042060 wound healing; panel c: combination of the two. NES: normalized enrichment score; NOM p value: nominal p value

**
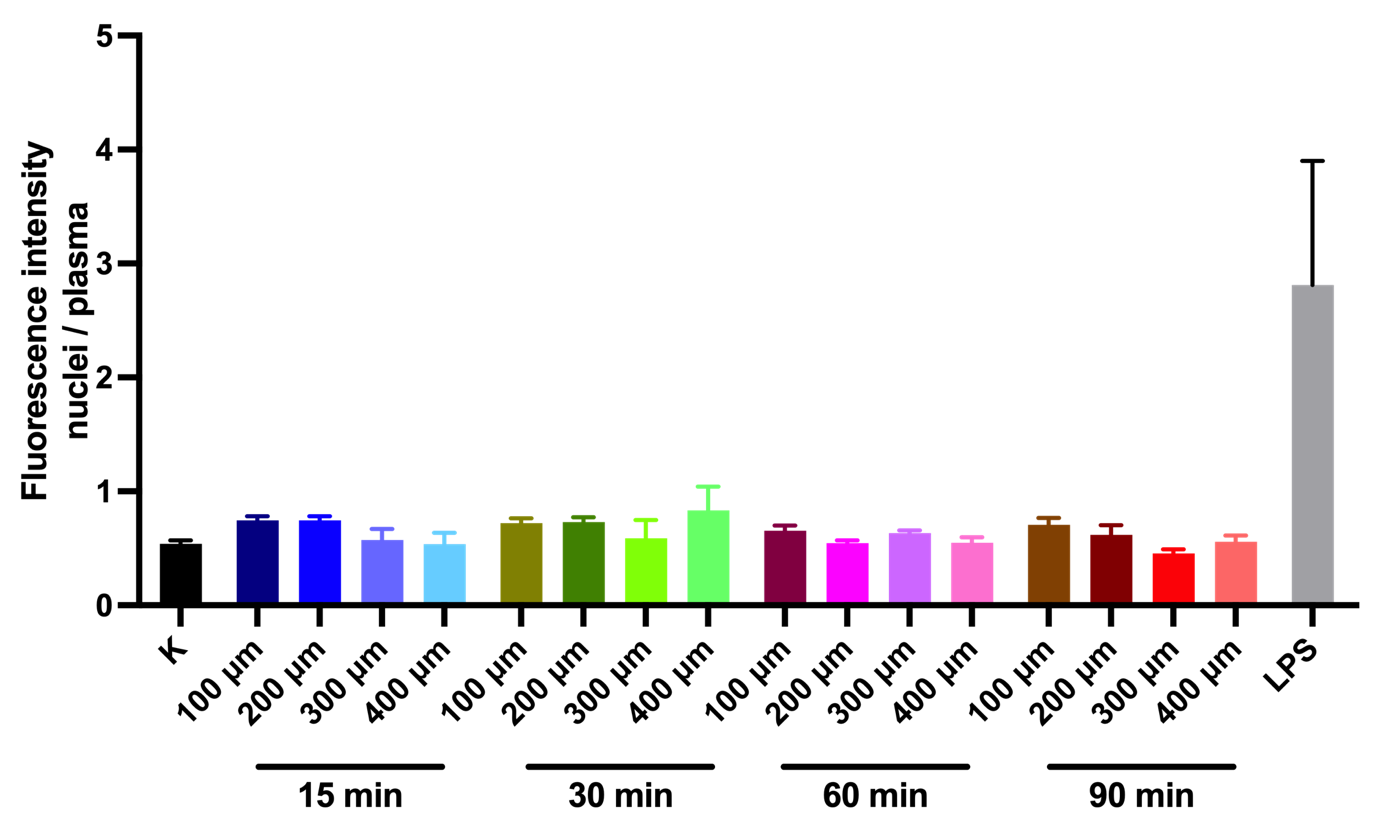
**

**Figure S2. Effect of mechanical wounding on NF-κB activation**

Confluent layers of HUVECs were cultured in 96-well plates and scratched using a sterile pipette tip to create a wound. Cells were fixed with ice-cold methanol-acetone (1:1) 15, 30, 60 or 90 minutes after scratching. We used 1 mg/ml LPS as a positive control. Cells were labelled with rabbit anti-human NFκB p65 antibody (1:200) and stained with goat anti-rabbit Alexa568 (1:500) and Hoechst (1:50000) nuclear staining. Images were taken using an Olympus IX-81 inverted fluorescence microscope and the ratio of cytoplasmic and nuclear mean red fluorescence was evaluated using CellP software. Distances were measured from the edge of the initial wound.

**Video S1. Mechanical wounding induces a Ca^2+^-wave on HUVECs**

Confluent layers of HUVECs were cultured in 96-well plates, cells were then loaded with 2 μM of Fluo-4-AM. Sequential images were taken every 5 seconds using a fluorescence microscope. Initially, two photographs were taken to determine baseline fluorescence, HUVEC layers were then scratched using a sterile pipette tip. The response was measured for 2 minutes, and then these images were subsequently converted to video.

**Video S2. Apyrase blocks propagation of mechanically induced Ca^2+^-wave on HUVECs**

Confluent layers of HUVECs were cultured in 96-well plates, cells were then loaded with 2 μM of Fluo-4-AM. Apyrase treatment (10 U/ml) was applied 5 minutes before measurement. Sequential images were taken every 5 seconds using a fluorescence microscope. Initially, two photographs were taken to determine baseline fluorescence, the HUVEC layers were then scratched using a sterile pipette tip. The response was measured for 2 minutes, these images were then subsequently converted to video.
