# Supplementary material for: Complement MASP-1 Modifies Endothelial Wound Healing
Source: Int J Mol Sci. 2024 Apr 5;25(7):4048. doi: 10.3390/ijms25074048 (PMC11012537; doi:10.3390/ijms25074048)
Supplement: Supplementary file 1 [file ijms-25-04048-s001.zip › supplementary material.pdf]

Complement MASP-1 modifies endothelial wound healing

Zsuzsanna Németh[1], Flóra Demeter[1], József Dobó[2], Péter Gál[2], László Cervenak[1,*]

[1] Research Laboratory, Department of Internal Medicine and Hematology, Semmelweis University, Budapest, Hungary
[2]Institute of Enzymology, HUN-REN Research Centre for Natural Sciences, Hungarian Research Network, Budapest, Hungary

Supplementary material

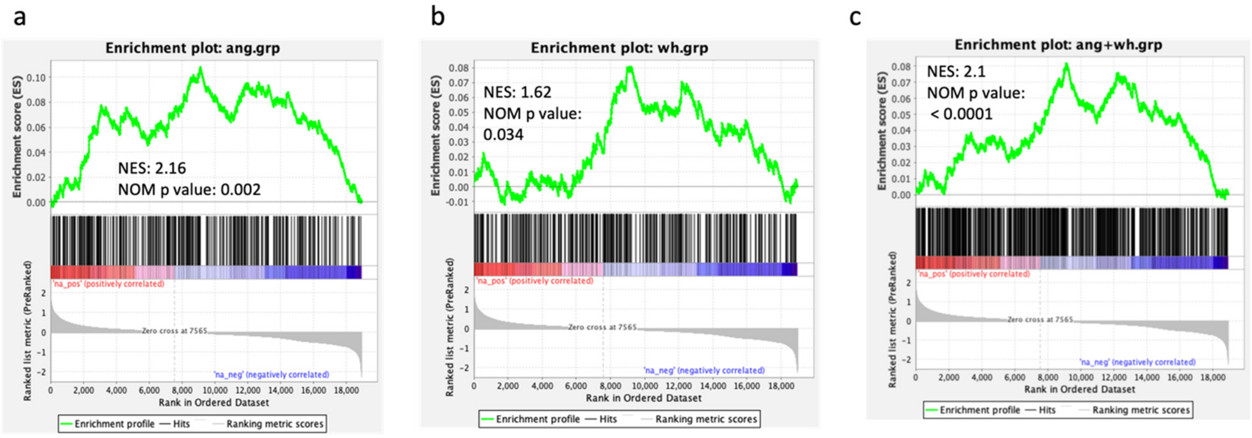

Figure S1. Gene set enrichment analysis (GSEA) showing significant enrichment of angiogenesis- and wound healing-related genes

GSEA were run on the database containing data from rMASP-1 treated and not treated HUVECs (available in the NBI Gene Expression Omnibus database, accession number: GSE98114). Genes were retrieved from the Gene Ontology Annotation database. Panel a: GO:0001525 angiogenesis; panel b: GO:0042060 wound healing; panel c: combination of the two. NES: normalized enrichment score; NOM p value: nominal p value

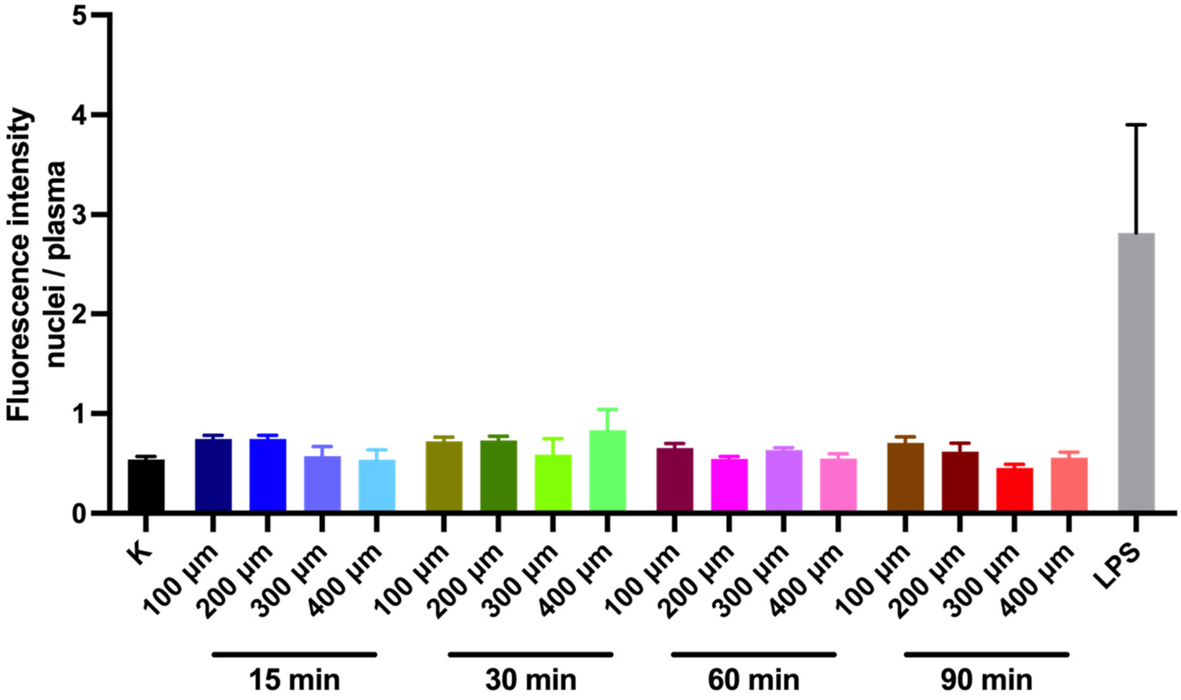

Figure S2. Effect of mechanical wounding on NF-κB activation

Confluent layers of HUVECs were cultured in 96-well plates and scratched using a sterile pipette tip to create a wound. Cells were fixed with ice-cold methanol-acetone (1:1) 15, 30, 60 or 90 minutes after scratching. We used 1 mg/ml LPS as a positive control. Cells were labelled with rabbit anti-human NFκB p65 antibody (1:200) and stained with goat anti-rabbit Alexa568 (1:500) and Hoechst (1:50000) nuclear staining. Images were taken using an Olympus IX-81 inverted fluorescence microscope and the ratio of cytoplasmic and nuclear mean red fluorescence was evaluated using CellP software. Distances were measured from the edge of the initial wound.

Video S1. Mechanical wounding induces a Ca^{2+}-wave on HUVECs

Confluent layers of HUVECs were cultured in 96-well plates, cells were then loaded with 2 µM of Fluo-4-AM. Sequential images were taken every 5 seconds using a fluorescence microscope. Initially, two photographs were taken to determine baseline fluorescence, HUVEC layers were then scratched using a sterile pipette tip. The response was measured for 2 minutes, and then these images were subsequently converted to video.

Video S2. Apyrase blocks propagation of mechanically induced Ca^{2+}-wave on HUVECs

Confluent layers of HUVECs were cultured in 96-well plates, cells were then loaded with 2 µM of Fluo-4-AM. Apyrase treatment (10 U/ml) was applied 5 minutes before measurement. Sequential images were taken every 5 seconds using a fluorescence microscope. Initially, two photographs were taken to determine baseline fluorescence, the HUVEC layers were then scratched using a sterile pipette tip. The response was measured for 2 minutes, these images were then subsequently converted to video.
