# Supplementary figures and images for: Complement MASP-1 Modifies Endothelial Wound Healing
Source: Int J Mol Sci. 2024 Apr 5;25(7):4048. doi: 10.3390/ijms25074048 (PMC11012537; doi:10.3390/ijms25074048)

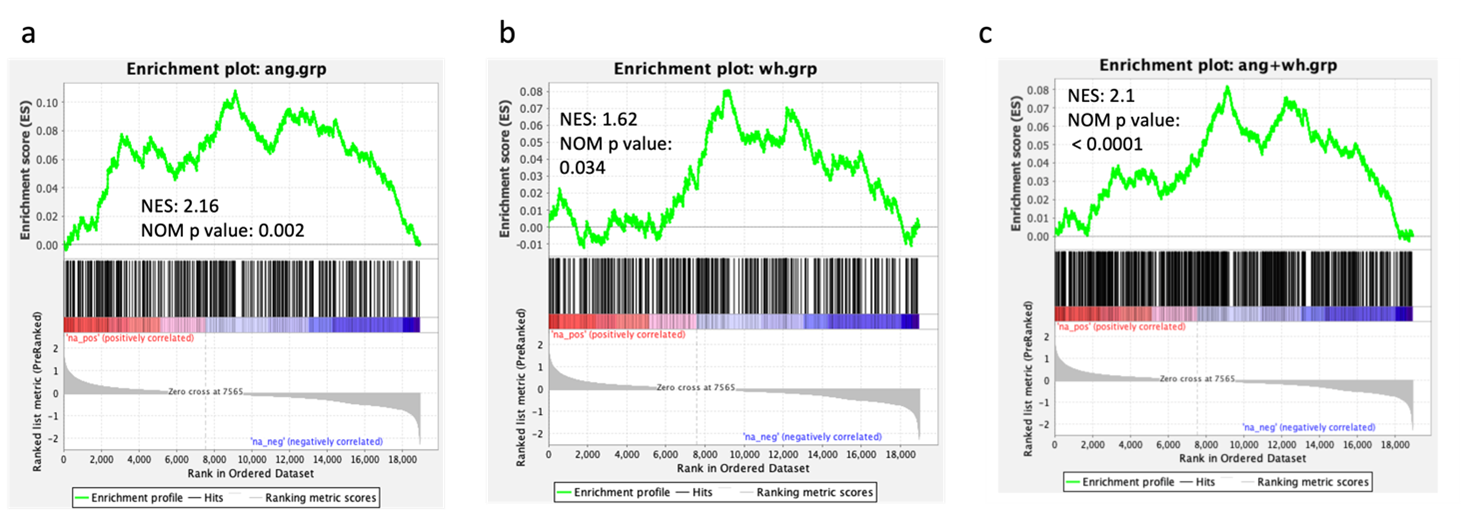

Supplement: Supplementary file 1 [file ijms-25-04048-s001.zip › Figure S1.png]

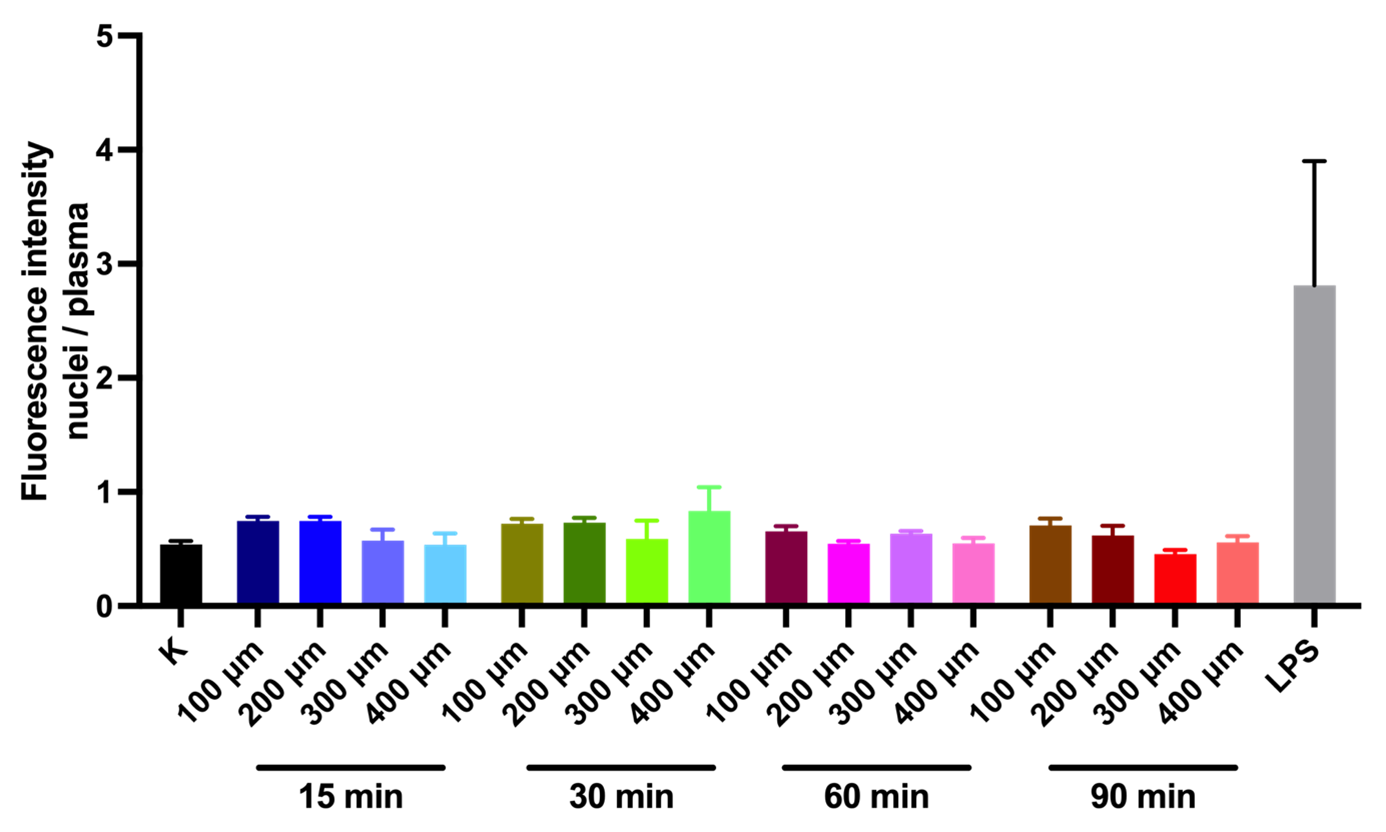

Supplement: Supplementary file 1 [file ijms-25-04048-s001.zip › Figure S2.png]
